# The Predictive Value of Pretreatment Lactate Dehydrogenase and Derived Neutrophil-to-Lymphocyte Ratio in Advanced Non-Small Cell Lung Cancer Patients Treated With PD-1/PD-L1 Inhibitors: A Meta-Analysis

**DOI:** 10.3389/fonc.2022.791496

**Published:** 2022-07-18

**Authors:** Qianning Zhang, Xiaoling Gong, Lei Sun, Liyun Miao, Yujie Zhou

**Affiliations:** ^1^ Department of Pharmacy, Nanjing Drum Tower Hospital, The Affiliated Hospital of Nanjing University Medical School, Nanjing, China; ^2^ School of Basic Medicine and Clinical Pharmacy, China Pharmaceutical University Nanjing Drum Tower Hospital, Nanjing, China; ^3^ Department of Respiratory and Critical Care Medicine, Nanjing Drum Tower Hospital, The Affiliated Hospital of Nanjing University Medical School, Nanjing, China

**Keywords:** lactate dehydrogenase, derived neutrophil-to-lymphocyte ratio, immunotherapy, non-small cell lung cancer, prognosis

## Abstract

**Background:**

The Lung Immune Prognostic Index (LIPI) combines the lactate dehydrogenase (LDH) level and the derived neutrophil-to-lymphocyte ratio (dNLR). A lot of studies have shown that LDH and dNLR are associated with the prognosis of advanced non-small cell lung cancer (NSCLC) in patients treated with programmed cell death protein 1 (PD-1) or programmed death-ligand 1 (PD-L1) inhibitors. However, previous results were inconsistent, and the conclusions remain unclear. This meta-analysis aimed to investigate the predictive value of pretreatment LDH and dNLR for NSCLC progression in patients treated with PD-1/PD-L1 inhibitors.

**Methods:**

PubMed, Embase, and the Cochrane Library were searched by two researchers independently for related literature before March 2020. Hazard ratios (HRs) with 95% confidence intervals (CIs) for progression-free survival (PFS) and overall survival (OS) were extracted to assess the predictive value of LDH and dNLR. STATA 15. 0 was used to perform the meta-analysis.

**Results:**

A total of 3,429 patients from 26 studies were included in this meta-analysis. The results revealed that high pretreatment LDH was related to poor OS (HR = 1.19, 95%CI = 1.11–1.24, *p* < 0.001), but not closely related to poor PFS (HR = 1.02, 95%CI = 1.00–1.04, *p* = 0.023 < 0.05). The pooled results for dNLR suggested that high pretreatment dNLR was related to poor OS (HR = 1.55, 95%CI = 1.33–1.80, *p* < 0.001) and PFS (HR = 1.33, 95%CI = 1.16–1.54, *p* < 0.001).

**Conclusion:**

Both pretreatment LDH and dNLR have the potential to serve as peripheral blood biomarkers for patients with advanced NSCLC treated with PD-1/PD-L1 inhibitors. However, more studies on LDH are needed to evaluate its predictive value for PFS in patients with NSCLC.

## Introduction

Lung cancer is a malignant tumor with the highest morbidity and mortality among all types of cancers globally (1). About 85% of patients with lung cancer were diagnosed with non-small cell lung cancer (NSCLC), and more than half of these patients were already in the advanced stage of cancer at the time of diagnosis. Drug therapy including chemotherapy, targeted drugs, and anti-angiogenic drugs can improve the prognosis of patients with NSCLC, but achieving long-term benefits is a challenge (2). The emergence of immune checkpoint inhibitors (ICIs) has been a huge breakthrough in the treatment of NSCLC, including programmed cell death protein 1 (PD-1) and programmed death-ligand 1 (PD-L1) inhibitors. These immune therapies significantly prolong the survival time of patients and have become one of the most essential treatments for advanced NSCLC. However, only a proportion of patients can benefit from ICIs, and others do not respond to immunotherapy, which limits their clinical application. Therefore, it is necessary to screen patients sensitive to immunotherapy in order to carry out the application of ICIs efficiently. PD-L1 expression and tumor mutational burden (TMB) are two popular biomarkers for predicting the efficacy of ICIs. PD-L1 is expressed in tumor cells and the surrounding microenvironment. When PD-1 is expressed in effector T cells binding to PD-L1, the activity of T cells can be suppressed, leading to the escape of tumor cells. Therefore, as the expression of PD-L1 is higher in tumor cells, the immunosuppressive effect of the PD-1/PD-L1 pathway becomes more active and treatment with ICIs becomes more effective. A higher TMB value means more mutated genes. Somatic mutations can lead to the expression of neoantigens, which can be recognized by T cells and can enhance the activity of ICIs. A lot of studies have shown that, in NSCLC, a high expression of PD-L1 is related to the efficacy of PD-1 inhibitors (3, 4). However, patients with negative PD-L1 expression can also benefit from ICIs (5–8). TMB has been recommended as a marker for NSCLC immunotherapy in the 2019 National Comprehensive Cancer Network (NCCN) guidelines, but there is no criterion to define a high level of TMB (9). Tissue biopsy needs to be conducted for the detection of PD-L1 and TMB, which requires high-quality specimens and is of high cost, but there is still a lack of uniform standard for detection platforms and measurement methods. In contrast, the acquisition of peripheral blood biomarkers is less invasive, low cost, and is easy to perform.

Inflammation is an essential component of the tumor microenvironment, promoting tumor proliferation, angiogenesis, metastasis, and destruction of adaptive immunity (10). The level of inflammation is related to the prognosis of malignant tumors. Tissue homeostasis can be perturbed by chronic inflammation, including local and systemic inflammation. Local inflammation includes tumor necrosis, inflammasomes, cytokines, chemokines, transcription factors, and tumor immune cell infiltrates, while systemic inflammation consists of circulating cytokines, small inflammatory proteins, circulating immune cells, and acute-phase proteins. The inflammatory factors coordinate the intracellular communication in the tumor microenvironment, and inflammatory conditions can disrupt the regulation of the innate and adaptive immune systems, leading to excessive tissue remodeling, loss of tissue architecture, protein alterations, and genotoxic DNA damage. These factors subsequently increase the risk of cancer and are crucial to distant tumor metastasis (11, 12). Inflammation-related index in serum, such as neutrophil count/lymphocyte count (NLR), lymphocyte count/monocyte count (LMR), and platelet count/lymphocyte count (PLR), can reflect the level of systemic inflammation. As opposed to a single peripheral blood index, the current trend is to combine multiple indexes to predict prognosis. The Lung Immune Prognostic Index (LIPI) is a predictive score that combines the lactate dehydrogenase (LDH) level and the derived neutrophil-to-lymphocyte ratio (dNLR) (13). Higher activity and expression level of LDH is closely related to the rapid proliferation of cancer cells through anaerobic glycolysis (14). The association between high pretreatment dNLR and poor prognosis was first reported in advanced melanoma patients treated with ICIs (15, 16), followed by studies on the prognostic value of dNLR for patients with NSCLC treated with immunotherapy. However, the association between dNLR and NSCLC prognosis was not significant in all related studies (17), and the role of dNLR in advanced NSCLC immunotherapy remains controversial.

In this study, we explore the potential predictive ability of LDH and dNLR and provide evidence for the prediction of LIPI. Therefore, we conducted this meta-analysis to examine the prognostic value of pretreatment serum dNLR and LDH levels in patients with advanced NSCLC receiving immunotherapy.

## Methods

### Search Strategy

We searched related literature systematically in databases including PubMed, Embase, and the Cochrane Library for studies published before March 2021. We performed both medical sub-heading (MeSH) terms and free words as a search strategy, and the main search terms included “non-small cell cancer”, “immune checkpoint inhibitors”, “programmed death-1 receptor”, “programmed death ligand-1”, “nivolumab”, “pembrolizumab”, “atezolizumab”, “durvalumab”, “avelumab”, “cemiplimab”, “camrelizumab”, “sintilimab”, “tislelizumab”, “toripalimab”, “lactate dehydrogenase”, “derived neutrophil-to-lymphocyte ratio”, “lung immune prognostic index”, “predictor”, “predict”, “prognosis”, “prognostic”, “peripheral blood biomarkers”, “serum biomarkers”, and “blood biomarkers”. The detailed search strategy is shown in **Supplementary Table S1**.

### Inclusion and Exclusion Criteria

The inclusion criteria were as follows: 1) research type: cohort study or case–control study; 2) object of research: patients with advanced NSCLC receiving PD-1 or PD-L1 treatment; 3) research purpose: the association between prognosis of NSCLC and LDH or dNLR; and 4) outcomes: available data on overall survival (OS) or progression-free survival (PFS) with the hazard ratio (HR) and 95% confidence interval (CI).

The exclusion criteria were as follows: 1) reviews, meta-analysis, conference abstract, and case report; 2) duplicate publication or overlap population; and 3) studies not written in English.

### Data Extraction

Two researchers screened the literature independently and extracted data, which included 1) basic information on the article: first author, publication year, country, sample size, follow-up time, study type, cutoff values for LDH and dNLR, and dNLR = neutrophil count to (white cell count − neutrophil count) (18); 2) patient characteristics: age, treatments, baseline LDH and NLR, and detection time; and 3) outcomes: association between LDH/dNLR and PFS/OS (the results of multivariate analysis were preferred, but those of univariate analysis were accepted if multivariate analyses were not reported). All data were cross-checked.

### Quality Assessment

Two researchers independently assessed the quality of the included studies according to the Newcastle–Ottawa Quality Scale (NOS). Studies with a score of 6 or higher were considered of high quality (19). Disagreement was resolved through discussion.

### Statistical Analysis

Meta-analysis was performed using STATA 15.0. We used HRs with 95% CIs to evaluate the relationship between dNLR/LDH and the prognosis of patients with advanced NSCLC. *I* ^2^ statistics and the *Q* test were used to assess the heterogeneity of the pooled results. When *p* < 0.1 and (or) *I* ^2^ > 50%, the heterogeneity is significant, and the random effects model was chosen for meta-analysis; otherwise, the fixed effects model was used. Subgroup analysis and sensitivity analysis were conducted to determine the sources of heterogeneity. Subgroup analysis was performed based on the region, analysis method, cutoff value, treatment, and sample size. Funnel plots and Egger’s test were conducted to assess publication bias. A *p* < 0.05 was considered statistically significant.

## Results

### Literature Search

**Figure 1:**
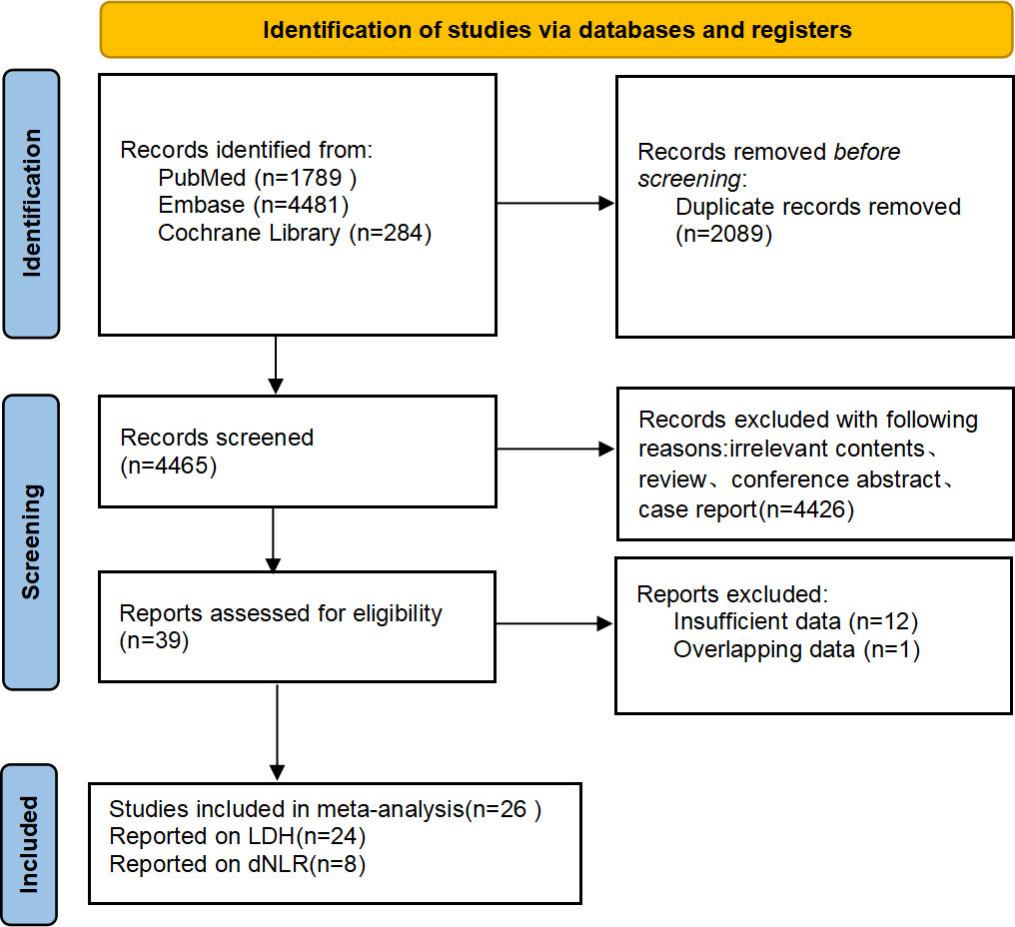
Flowchart of the included studies.

According to the established search strategy, 6,554 studies were retrieved from the databases. We excluded 2,089 duplicate studies and 4,425 unrelated literature. A total of 40 studies were chosen for further review. Finally, 26 retrospective studies were included, of which 24 studies reported the relationship between LDH and the prognosis of patients with NSCLC treated with immunotherapy and 8 studies reported the association between dNLR and prognosis. The flowchart of literature selection is shown in **Figure 1**.

### Characteristics of the Included Literature and Quality Assessment

All included studies were published between 2017 and 2020, and the sample size was between 20 and 466. Nine studies were conducted in European countries, 15 were carried out in Asia, and only 1 was conducted in Australia. The cutoff values for LDH and dNLR differed, ranging from 207 to 400 U/L and from 2.2 to 3, respectively. In terms of treatment, except for three studies that incorporated patients treated with ICI combination therapy, the studies only included patients who received a single treatment of ICIs. Based on the NOS, quality assessment of the included studies revealed that all of them were considered of high quality, with a score of 6 or higher. The basic characteristics of the included studies are shown in **Table 1**.

**Table 1:** Characteristics of the included studies.

Reference	Study period	Year	Region	Study design	Follow-up	Sample size	Age (years)	Treatment	Collection time (days)	LDH cutoff (U/L)	dNLR cutoff	Outcomes	NOS
Hasegawa et al. (20)	2017–2019	2019	Japan	R	9.5 (0.5–25.6)	51	70 (35–86)	Pemb mono	7	222	NR	PFS, OS	6
Mezquita et al. (21)	2012–2017	2018	France	R	12 (11–14)	466	62 (29–86)	PD-1/PD-L1/CTLA-4–PD-L1 mono/combined	NR	ULN	3	OS	8
Svaton et al. (22)	2015–2016	2018	Czech Republic	R	NR	70	NR	Nivo mono	1	ULN	NR	PFS, OS	6
Yuan et al. (23)	2017–2020	2020	China	R	NR	92	65 (55–70)	Nivo, Pemb, Sint, Tisle mono	NR	207	2.38	PFS, OS	7
Giannicola et al. (24)	2015–2018	2019	Italy	R	9	92	66	Nivo mono	NR	NR	NR	PFS, OS	6
Lang et al. (25)	2015–2018	2019	Austria	R	NR	84	69 (46–90)	Nivo, Pemb, Atezo mono	NR	248	NR	PFS, OS	6
Ruiz-Bañobre et al. (26)	2015–2017	2019	Spain	R	NR	153	63	Nivo mono	30	ULN	NR	PFS	7
Lobefaro et al. (27)	2013–2020	2021	Italy	R	29 (IQR = 13.38–47.41)	310	65.7 (27–88)	Nivo, Pemb, Atezo mono/combined	7	400	NR	PFS, OS	8
Tamiya1 et al. (28)	2015–2016	2020	Japan	R	NR	201	68 (27–87)	Nivo mono	NR	214	NR	OS	7
Tanizaki et al. (29)	2015–2016	2017	Japan	R	NR	134	68 (33–85)	Nivo mono	7	222	NR	PFS, OS	7
Peng et al. (30)	2017–2019	2019	China	R	NR	102	62	Nivo, Pemb, Sint, Tori mono	7	240	NR	PFS, OS	7
Ichiki et al. (31)	2016–2018	2018	Japan	R	4.8	44	71 (42–91)	Nivo, Pemb mono	NR	NR	NR	OS	7
Takada et al. (32)	2016–2018	2020	Japan	R	13.7 (0–43.8)	226	66 (31–88)	PD-1 mono	NR	350	2.79	PFS, OS	7
Mazzaschi et al. (33)	2015–2019	2020	Italy	R	17.3	109	72 (41–85)	Nivo, Pemb, Atezo mono	NR	248	3	PFS, OS	8
Kataoka et al. (34)	2016	2018	Japan	R	NR	189	69 (38–88)	Nivo mono	1	217	NR	PFS	6
Katayama et al. (35)	2018–2019	2020	Japan	R	NR	81	71 (42–84)	Atezo mono	10	227	NR	PFS, OS	6
Diem et al. (36)	2015–2016	2017	Switzerland	R	0–14	52	66 (46–88)	Nivo mono	7	246	NR	PFS, OS	6
Sakata et al. (37)	2016–2018	2019	Japan	R	14.2 (21–36.3)	83	69 (42–83)	Nivo, Pemb, Atezo mono	NR	223	NR	OS	8
Tamiya et al. (38)	2017–2018	2019	Japan	R	11	51	70 (35–86)	Pemb mono	NR	222	NR	PFS	8
Adachi et al. (39)	2015–2018	2019	Japan	R	26.6	296	70 (64–76)	Nivo mono	NR	240	NR	PFS	8
Seban et al. (40)	2017–2019	2020	France	R	13.4	63	65 (37–86)	Pemb mono	NR	ULN	3	PFS, OS	6
Huang et al. (41)	2016–2018	2020	China	R	NR	61	57 (20–75)	PD-1/PD-L1/CTLA-4–PD-L1 mono/combined	NR	369	NR	PFS, OS	6
Oya et al. (42)	2015–2017	2017	Japan	R	6 (0.1–22.5)	124	66 (37–79)	Nivo mono	1	245	NR	PFS	7
Prelaj et al. (43)	2015–2018	2019	Italy	R	NR	154	67 (31–86)	Nivo, Pemb mono	14	NR	2.2	PFS, OS	7
Mirili et al. (44)	2015–2019	2019	Turkey	R	9 (1–44)	20	61 (41–74)	Nivo mono	NR	NR	1.9	OS	7
Zhai et al. (45)	2017–2019	2020	China	R	11.4 (2.1–24.7)	121	62 (29–81)	PD-1/PD-L1/CTLA-4–PD-L1 mono/combined	30	ULN	3	PFS, OS	8

R, retrospective; NR, not reported; LDH, lactate dehydrogenase; dNLR, derived neutrophil-to-lymphocyte ratio; Nivo, nivolumab; Pemb, pembrolizumab; Atezo, atezolizumab; Sint, sintilimab; Tori, toripalimab; PD-1, programmed cell death protein 1 inhibitor; PD-L1, programmed death-ligand 1 inhibitor; CTLA-4, cytotoxic T-lymphocyte-associated protein 4 inhibitor; mono, monotherapy; combined, combined therapy; ULN, upper limit of normal; HR, hazard ratio; OS, overall survival; PFS, progression-free survival.

### Results of LDH

#### Impact of LDH on OS and PFS

**Figure 2:**
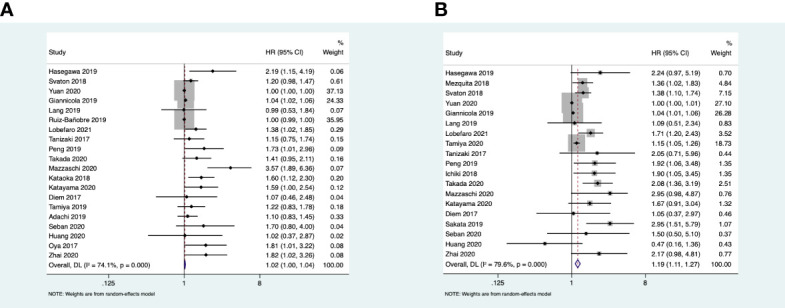
**(A)** Forest plots of the association between pretreatment lactate dehydrogenase (LDH) and progression-free survival. **(B)** Forest plots of the association between pretreatment LDH and overall survival.

Nineteen studies reported the association between LDH and OS, and the heterogeneity between the studies was apparent (*I* ^2^ = 79.6% > 50%; *Q* test, *p* < 0.1). Therefore, the random effects model was adopted. The results of the meta-analysis showed that a high baseline LDH was related to poor OS (HR = 1.19, 95%CI = 1.11–1.24, *p* < 0.001). Twenty studies reported the association between LDH and PFS with an apparent heterogeneity (*I* ^2^ = 74.1% > 50%; *Q* test, *p* < 0.1); we still used the random effects model. The pooled results showed that a high baseline LDH was weakly correlated with poor PFS (HR = 1.02, 95%CI = 1.00–1.04, *p* = 0.023 < 0.05) (**Figure 2**).

#### Subgroup Analysis

To further evaluate the predictive value of LDH, we performed subgroup analysis based on the region, analysis method, cutoff value, treatment, and sample size. The Australian subgroup and the combination treatment group did not show any correlation to LDH and OS. For PFS, the European and Australian subgroups, a sample size <100, and the multivariate analysis did not show a significant association. As there were differences among the studies reporting the cutoff values, we used the median value for the subgroup analysis. In the PFS subgroup analysis, the data showed that the pooled HR was 1.44 (95%CI = 1.14–1.81) when the cutoff value of LDH was ≥ 240 U/L and was 1.32 (95%CI = 1.03–1.69) when the cutoff value was <240 U/L. In the OS subgroup analysis, the data showed that the pooled HR was 1.61 (95%CI = 1.17–2.21) when the cutoff value of LDH was ≥ 240 U/L and 1.25 (95%CI = 1.03–1.50) when the cutoff value was <240 U/L (**Table 2**).

**Table 2:** Subgroup analysis.

Analysis	PFS					OS				
	Study	Association		Heterogeneity		Study	Association		Heterogeneity	
		HR (95%CI)	*p*-value	*I* ^2^	*p*-value		HR (95%CI)	*p*-value	*I* ^2^	*p*-value
LDH										
	20					19				
Region										
Europe	7	1.05 (1.00–1.11)	0.065	85.30%	<0.001	7	1.38 (1.09–1.75)	0.008	74.20%	0.001
Asia	12	1.35 (1.14–1.62)	0.001	66.10%	0.001	11	1.46 (1.20–1.76)	<0.001	80.80%	<0.001
Australia	1	0.99 (0.53–1.84)	0.975	–	–	1	1.09 (0.51–2.34)	0.833	–	–
Analysis										
Univariate	11	1.17 (1.04–1.31)	0.01	29.00%	0.17	9	1.33 (1.04–1.71)	0.026	66.60%	0.002
Multivariable analysis	9	1.01 (0.99–1.02)	0.471	80.90%	<0.001	10	1.46 (1.22–1.75)	<0.001	82.90%	<0.001
Treatment										
Monotherapy	17	1.02 (1.00–1.03)	0.036	75.30%	<0.001	15	1.14 (1.07–1.23)	<0.001	79.90%	<0.001
Monotherapy + combination therapy	3	1.43 (1.11–1.85)	0.006	0.00%	0.567	4	1.43 (0.99–2.06)	0.055	52.10%	0.1
Sample size										
<100	10	1.04 (1.00–1.08)	0.086	68.50%	0.001	11	1.07 (1.00–1.13)	0.043	74.10%	<0.001
>100	10	1.45 (1.16–1.80)	0.001	79.40%	<0.001	8	1.65 (1.29–2.12)	<0.001	66.90%	0.004
LDH cutoff										
≥240 U/L	9	1.44 (1.14–1.81)	0.002	47.60%	0.054	7	1.61 (1.17–2.21)	0.003	43.00%	0.104
<240 U/L	6	1.32 (1.03–1.69)	0.029	71.00%	0.004	6	1.25 (1.03–1.50)	0.021	81.00%	<0.001
dNLR										
	5					7				
Region										
Europe	3	1.51 (1.19–1.91)	0.001	0.00%	0.617	4	1.59 (1.32–1.91)	<0.001	24.10%	0.267
Asia	3	1.24 (1.04–1.48)	0.001	58.50%	0.09	4	1.48 (1.16–1.89)	0.002	0.00%	0.828
Sample size										
<100	2	1.30 (1.06–1.58)	0.01	47.40%	0.168	4	1.51 (1.24–1.84)	<0.001	2.00%	0.382
>100	4	1.33 (1.16–1.54)	0.002	44.30%	0.145	4	1.60 (1.28–1.99)	<0.001	0.00%	0.603
Treatment										
Monotherapy	5	1.38 (1.19–1.59)	<0.001	0.00%	0.581	6	1.51 (1.27–1.81)	<0.001	0.00%	0.531
Monotherapy + combination therapy	1	0.65 (0.33–1.27)	0.21	–	–	2	1.63 (1.24–2.15)	0.001	0.00%	0.395
dNLR cutoff										
≥3	3	1.38 (1.10–1.74)	0.005	0.00%	0.602	4	1.60 (1.33–1.92)	<0.001	13.70%	0.324
<3	3	1.30 (1.09–1.56)	0.004	68%	0.044	4	1.47 (1.14–1.88)	0.002	0.00%	0.734

PFS, progression-free survival; LDH, lactate dehydrogenase; dNLR, derived neutrophil-to-lymphocyte ratio.

#### Sensitivity Analysis

We performed a sensitivity analysis to determine the source of heterogeneity and explore the impact of every single study on the overall effect. In the OS analysis, the results showed that the study of Yuan et al. and that of Ginanicola et al. had greater impact on the overall analysis, and the heterogeneity was significantly obvious. After excluding the study of Yuan et al., the heterogeneity was *I* ^2^ = 73.9% (*Q* test, *p* < 0.1) and the pooled HR = 1.47 (95%CI = 1.27–1.70, *p* < 0.001). When we excluded the study of Giannicola et al., the heterogeneity was *I* ^2^ = 78.8% (*p* < 0.001) and the pooled HR = 1.48 (95%CI = 1.26–1.73, *p* < 0.001). In the analysis on PFS, the studies of Yuan et al., Ruiz et al., and Giannicola et al. were highly heterogeneous. When the study of Yuan was removed, the heterogeneity was *I* ^2^ = 75.4% (*Q* test, *p* < 0.001) and the combined HR = 1.12 (95%CI = 1.06–1.19, *p* < 0.001). When the study of Ruiz et al. was removed, *I* ^2 =^ 74.9% (*Q* test, *p* < 0.1) and the combined HR = 1.12 (95%CI = 1.06–1.19, *p* < 0.001). After excluding the study of Giannicola et al., *I* ^2^ = 69.8% (*Q* test, *p* < 0.1) and the combined HR = 1.01 (95%CI = 0.99–1.03, *p* = 0.234). Since the excessively high heterogeneity can be attributed to several studies, it cannot be reduced by excluding only one of these studies. In the PFS analysis, the combined result was different from the original one when we excluded the study of Giannicola et al. (HR = 1.01, 95%CI = 0.99–1.03, *p* = 0.234). However, the results were consistent with the initial results after excluding any other highly heterogeneous studies. Thus, we hypothesized that the heterogeneity of these studies did not affect the meta-analysis results, except for that of Giannicola et al. (**Supplementary Figure S1**).

#### Publication Bias

Funnel charts were drawn and Egger’s test was performed to assess publication bias. Both the asymmetric funnel charts and Egger’s test indicated a high risk of potential publication bias regarding the HRs of OS (*p* < 0.001) and PFS (*p* < 0.001). Studies with a small sample size may have a high risk of potential publication bias (**Supplementary Figures S2, S3**).

### Results of dNLR

#### Impact of dNLR on OS and PFS

**Figure 3:**
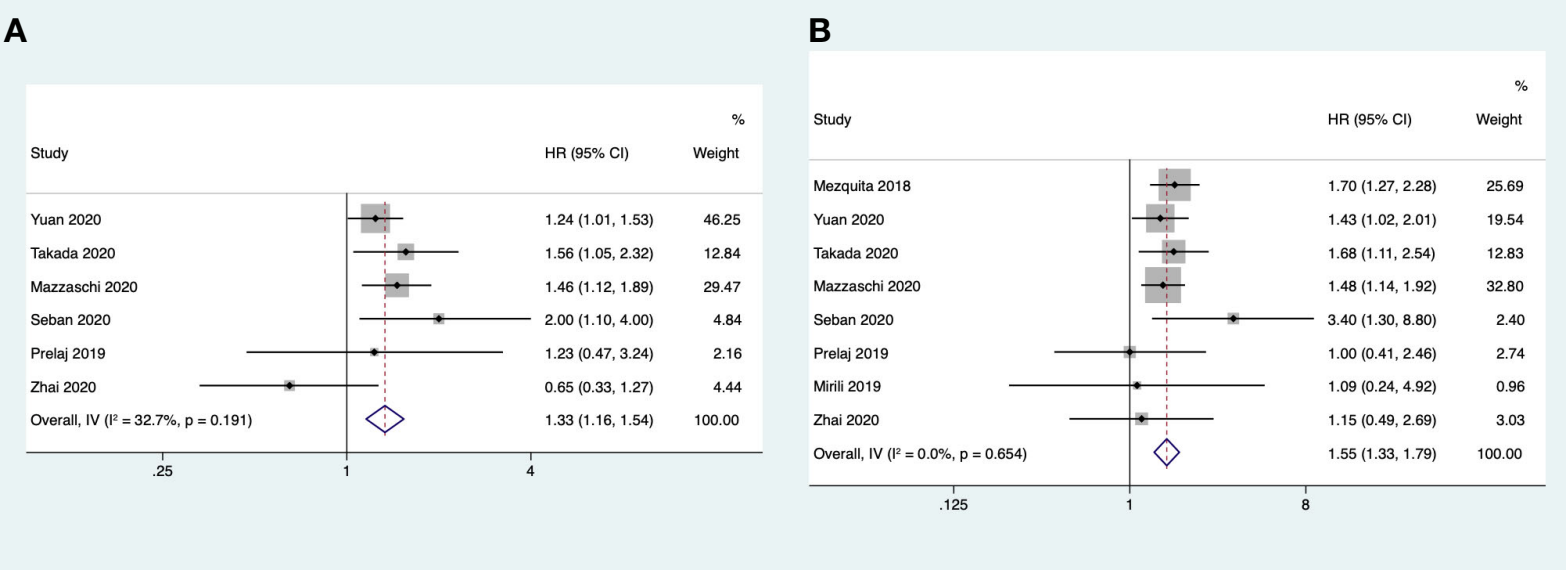
**(A)** Forest plots of the association between pretreatment derived neutrophil-to-lymphocyte ratio (dNLR) and progression-free survival. **(B)** Forest plots of the association between pretreatment dNLR and overall survival.

Eight studies reported the association between dNLR and OS, but no significant heterogeneity was found among these studies (*I* ^2^ = 0% < 50%; *Q* test, *p* = 0.654 > 0.1). We therefore used the fixed effects model for analysis. The meta-analysis results showed that a high baseline dNLR value was associated with poor OS (HR = 1.55, 95%CI = 1.33–1.80, *p* < 0.001). Six studies reported the association between dNLR and PFS, where the heterogeneity was not significant (*I* ^2^ = 32.7% < 50%; *Q* test, *p* = 0.19 > 0.1) and the fixed effects model was used. The combined results showed that a high baseline dNLR value was associated with poor PFS (HR = 1.33, 95%CI = 1.16–1.54, *p* < 0.001) (**Figure 3**).

#### Subgroup Analysis

The subgroup analysis for OS and PFS was conducted by region, sample size, cutoff value = 3, and treatment. Only the studies on combination therapy were found to have no correlation with PFS. The other subgroups were all correlated to poor OS and PFS. In the PFS subgroup analysis, the data showed that the pooled HR was 1.38 (95%CI = 1.10–1.74) when the cutoff value was ≥ 3 and was 1.30 (95%CI = 1.09–1.56) when the cutoff was <3. In the OS subgroup analysis, the data showed that the pooled HR was 1.60 (95%CI = 1.33–1.92) when the cutoff value was ≥ 3 and 1.47 (95%CI = 1.14–1.88) when the cutoff value was <3 (**Table 2**).

#### Publication Bias

The funnel charts were symmetrical, and Egger’s test suggested no potential publication bias regarding the HRs of OS (*p* = 0.857) and PFS (*p* = 0.891) (**Supplementary Figures S2, S3**).

## Discussion

More and more studies have shown that systemic inflammation plays a vital role in the occurrence, development, and metastasis of tumors (46). LDH is a classic inflammatory index. The Warburg effect is one of the crucial characteristics of tumor cells: glycolysis occurs in aerobic conditions, and LDH catalyzes pyruvate to produce a large amount of lactic acid, which provides energy for tumor growth (47). The low pH and hypoxic microenvironment promote the proliferation of immunosuppressive cells, and LDH is related to the activation of the vascular endothelial growth factor (VEGF) pathway and the stimulation of tumor angiogenesis (48). Various pieces of evidence have indicated the promotional role of LDH in tumor growth. Neutrophils remodel the extracellular matrix by releasing specific proteases and then migrate into the remodeled extracellular matrix and tissues to form a new vascular supply, thereby promoting the migration of tumor-associated endothelial cells in new blood vessels (49). Lymphocytes, such as tumor-infiltrating lymphocytes (TILs), play an essential role in antitumor immunity and also function as a good prognostic indicator in many cancer types (50). A meta-analysis reported by Zhang et al. evaluated the correlation between NLR and the prognosis of patients with advanced NSCLC treated with immunotherapy (51). Both formulas for NLR and dNLR used the neutrophil count value as the numerator, but their denominators differed. The formula for NLR used the lymphocyte count value as the denominator. However, in terms of dNLR, the value for the white blood cell count minus the neutrophil count in the denominator was similar to that of the lymphocyte count. There may be a decrease in the lymphocyte count and an increase in the monocyte count in patients with cancer. NLR was unable to take the effect of monocytes into account. However, in the formula for dNLR, the denominator mixed two cell types, namely, lymphocytes and monocytes, with possible contradictory effects on the predictive value (18).

This meta-analysis included 26 studies involving a total of 3,429 patients with advanced lung cancer treated with ICIs. A systematic analysis was conducted to reveal that elevated pretreatment dNLR is associated with poorer PFS and OS. Considering that the first PD-1 inhibitors went on the market in China in 2018, many new studies have investigated the predictive role of LDH in recent years. This study also further verified that the high pretreatment LDH level is related to poor OS, which is consistent with the results of a previous study (52). The pooled results of the multivariate analysis showed that LDH is not an independent prognostic indicator of PFS, which is inconsistent with the results of a previous study (52). However, in the LDH subgroup analysis based on region, patient cohorts from Australia did not show a correlation with PFS or OS, which may be due to there being only one article included and its small sample size. Patient cohorts from Asia showed an association with OS and PFS, while the studies from Europe did not show any correlation to PFS, but were correlated with OS. The differences in the effect of LDH on the prognosis of immunotherapy among races need to be explored with more clinical research. There is still no uniform standard to define the cutoff value of LDH, and the definition of the cutoff in each study differed. Although LIPI clearly defines LDH ≥  ULN as one of the risk factors, the definition of ULN in each research center still differed (21). In this study, the values “3” and “240” were defined as the cutoff for dNLR and LDH, respectively, to perform subgroup analysis. The results showed that both dNLR ≥  3 and dNLR < 3 were associated with poor PFS and OS, and the risk of LDH ≥  240 U/L and LDH < 240 U/L was significantly higher in both PFS and OS. However, more high-quality research is still needed to determine the critical standard for the cutoff values. In this meta-analysis, only four studies involved patients receiving combination treatments. The subgroup analysis conducted based on treatment showed that baseline dNLR did not correlate with poor PFS and that baseline LDH was not associated with poor OS in studies on combination therapy. Current studies have mainly focused on the prognosis of immune monotherapy for NSCLC. However, there are multiple clinical trials of immune combination therapy (7, 53, 54), ICI combination chemotherapy, anti-angiogenic drugs, or double immunotherapy, which are the clinical practice trends. Two retrospective studies explored the predictive value of LIPI for patients treated with ICIs. Wang et al. hypothesized that LIPI is the only prognostic indicator of ICI monotherapy, which was shown to have no correlation with the prognosis of ICIs combined with chemotherapy (55). In contrast, Blanc-Durand et al. found that pretreatment LIPI correlated with ICI survival in monotherapy and in combination with chemotherapy (56). The predictive value of peripheral blood inflammation indicators for immune combination therapy is still unclear.

Our study had some limitations. Firstly, all included articles were retrospective studies with selection bias, and the sample size of each study comparatively differed. Secondly, the combined results for LDH were highly heterogeneous and showed significant publication bias. Thirdly, some of the ICIs were approved only for patients with a positive PD-L1 expression, such as pembrolizumab, atezolizumab, and cemiplimab. In these cases, detection of the PD-L1 tumor proportion score (TPS) is unavoidable, and PD-L1 tumor staining combined with the blood values may underline the essential role of tumor inflammation and its microenvironment. In a multicenter retrospective exploratory analysis, patients with NLR <5 + PD-L1 >80% obtained better 2-year OS and PFS compared with patients with NLR >5 (OS: HR = 0.20, *p* = 0.006; PFS: HR = 0.44, *p* = 0.03). This result revealed that the TPS of PD-L1 combined with the blood values showed a stronger prognostic role (57). Fourthly, our meta-analysis did not include studies conducted in the United States and Africa, which leads to the lack of comprehensiveness. We did not find studies published in these countries that met our study inclusion criteria, which may be caused by search bias. Finally, this study only considered the impact of the levels of dNLR and LDH before treatment on the prognosis and their dynamic changes were not taken into consideration. A retrospective analysis investigated the early discrepancies in the NLR and dNLR in patients with NSCLC treated with ICI therapy. The results showed the PFS to be significantly shorter in the group with increased NLR than in the non-increased NLR group (2.6 *vs*. 9.5 months, *p* < 0.001), and the same result was found in the dNLR group (4.2 *vs*. 9.2 months, *p* = 0.001) (58). Another team explored changes in the Gustave Roussy immune score (GRIm-score) in 45 days, which comprised the NLR, LDH, and serum albumin concentration. Their results suggested that a low GRIm-score in the 45-day group achieved significantly longer PFS and OS than the high-score group. Patients with stable/positive GRIm-score changes had better OS, PFS, and overall response rate (ORR) than did patients with negative changes in their GRIm-score (59). These studies indicated that early changes in the inflammatory blood values might be more reliable predictors of prognosis than pretreatment blood biomarkers. It could be the basis for treatment modifications by monitoring dynamic changes in inflammatory blood markers.

In summary, this study demonstrated that a high baseline LDH is significantly associated with poor OS and that a high baseline dNLR is significantly associated with both poor OS and PFS in patients with NSCLC treated with ICIs. Baseline LDH and dNLR values could be convenient and affordable predictive biomarkers, which can select and stratify patients who could benefit from ICIs, promoting the development of individualized therapy. More well-designed and large-scale studies are still needed to confirm these results.

## Data Availability Statement

The original contributions presented in the study are included in the article/**Supplementary Material**, further inquiries can be directed to the corresponding authors.

## Author Contributions

YZ and LM had full access to all of the data in the study and take responsibility for the integrity of the data and the accuracy of the data analysis. QZ, YZ, and LM: Concept and design. All authors: Acquisition, analysis, or interpretation of data. QZ, YZ, and LM: Drafting of the manuscript. QZ, XG, LS, and LM: Critical revision of the manuscript for important intellectual content. QZ: Statistical analysis. YZ and LM: Administrative, technical, or material support and study supervision. All authors contributed to the article and approved the submitted version.

## Conflict of Interest

The authors declare that the research was conducted in the absence of any commercial or financial relationships that could be construed as a potential conflict of interest.

## Publisher’s Note

All claims expressed in this article are solely those of the authors and do not necessarily represent those of their affiliated organizations, or those of the publisher, the editors and the reviewers. Any product that may be evaluated in this article, or claim that may be made by its manufacturer, is not guaranteed or endorsed by the publisher.
