# Supplementary material for: The Predictive Value of Pretreatment Lactate Dehydrogenase and Derived Neutrophil-to-Lymphocyte Ratio in Advanced Non-Small Cell Lung Cancer Patients Treated With PD-1/PD-L1 Inhibitors: A Meta-Analysis
Source: Front Oncol. 2022 Jul 18;12:791496. doi: 10.3389/fonc.2022.791496 (PMC9340347; doi:10.3389/fonc.2022.791496)
Supplement: Supplementary file 1 [file DataSheet_1.docx]

**Supplementary Online Content**

**Supplementary table 1.** Search Strategies for PubMed, Embase and Cochrane Library

**Supplementary figure 1.** (A)Sensitivity analysis on the relationship between pretreatment LDH and PFS in advanced NSCLC; (B)Sensitivity analysis on the relationship between pretreatment LDH and OS in advanced NSCLC.

**Supplementary figure 2.** Funnel Plot of the relationship between(A) pretreatment LDH and PFS in advanced NSCLC,(B)pretreatment LDH and OS in advanced NSCLC,(C) pretreatment dNLR and PFS in advanced NSCLC,(D)pretreatment dNLR and OS in advanced NSCLC.

**Supplementary figure 3.** Egger’s test of the relationship between (A) pretreatment LDH and PFS in advanced NSCLC,(B)pretreatment LDH and OS in advanced NSCLC,(C) pretreatment dNLR and PFS in advanced NSCLC,(D)pretreatment dNLR and OS in advanced NSCLC.

**Supplementary table 1**. Search Strategies for PubMed, Embase and Cochrane Library

| **Database** | **Keywords** |
| --- | --- |
| **PubMed** | 1789 |
| #1 | "carcinoma, non small cell lung"[MeSH Terms] |
| #2 | "carcinoma non small cell lung"[Title/Abstract] OR "carcinomas non small cell lung"[Title/Abstract] OR "lung carcinoma non small cell"[Title/Abstract] OR "lung carcinomas non small cell"[Title/Abstract] OR "non small cell lung carcinomas"[Title/Abstract] OR "non small cell lung carcinoma"[Title/Abstract] OR "non small cell lung carcinoma"[Title/Abstract] OR "carcinoma non small cell lung"[Title/Abstract] OR "non small cell lung carcinoma"[Title/Abstract] OR "non small cell lung cancer"[Title/Abstract] OR "nonsmall cell lung cancer"[Title/Abstract] |
| #3 | #1 OR #2 |
| #4 | "immune checkpoint inhibitor"[Title/Abstract] OR "Immunotherapy"[Title/Abstract] OR "ICI"[Title/Abstract] OR "PD-1"[Title/Abstract] OR "programmed cell death 1 protein"[Title/Abstract] OR "PD-L1"[Title/Abstract] OR "programmed cell death 1 receptor"[Title/Abstract] OR "pembrolizumab"[Title/Abstract] OR "nivolumab"[Title/Abstract] OR "atezolizumab"[Title/Abstract] OR "durvalumab"[Title/Abstract] OR "avelumab"[Title/Abstract] OR "Cemiplimab"[Title/Abstract] OR "Camrelizumab"[Title/Abstract] OR "Sintilimab"[Title/Abstract] OR "Tislelizumab"[Title/Abstract] OR "Toripalimab"[Title/Abstract] |
| #5 | “Lactate Dehydrogenase”[All Fields] OR “LDH”[All Fields] OR “derived neutrophil-lymphocyte ratio”[All Fields] OR “derived neutrophil-to-lymphocyte ratio”[All Fields] OR “dNLR”[All Fields] OR “Lung Immune Prognostic Index”[All Fields] OR “LIPI”[All Fields] OR “Peripheral Blood Biomarkers[Title/Abstract]” OR “serum biomarkers[Title/Abstract]”” OR “Predictive[Title/Abstract]” OR “predict[Title/Abstract]” OR “prognosis[Title/Abstract]” OR “prognostic[Title/Abstract]” |
| #6 | #3 AND #4 AND #5 |
| **Embase** | 4481 |
| #1 | 'non small cell lung cancer'/exp |
| #2 | 'carcinoma, non small cell lung':ab,ti OR 'carcinomas, non-small-cell lung':ab,ti OR 'lung carcinoma, non-small-cell':ab,ti OR 'lung carcinomas, non-small-cell':ab,ti OR 'non-small-cell lung carcinomas':ab,ti OR 'non-small-cell lung carcinoma':ab,ti OR 'non small cell lung carcinoma':ab,ti OR 'carcinoma, non-small cell lung':ab,ti OR 'non-small cell lung carcinoma':ab,ti OR 'non-small cell lung cancer':ab,ti OR 'nonsmall cell lung cancer':ab,ti |
| #3 | #1 OR #2 |
| #4 | 'immune checkpoint inhibitor':ab,ti OR 'ici':ab,ti OR 'pd-1':ab,ti OR 'programmed cell death 1 protein':ab,ti OR 'pd-l1':ab,ti OR 'programmed cell death 1 receptor':ab,ti OR 'immunotherapy':ab,ti OR 'pembrolizumab':ab,ti OR 'nivolumab':ab,ti OR 'atezolizumab':ab,ti OR  'durvalumab':ab,ti OR 'avelumab':ab,ti OR 'cemiplimab':ab,ti OR 'camrelizumab':ab,ti OR 'sintilimab':ab,ti OR 'tislelizumab':ab,ti OR 'toripalimab':ab,ti |
| #5 | 'lactate dehydrogenase' OR 'ldh' OR 'derived neutrophil-lymphocyte ratio' OR 'derived neutrophil-to-lymphocyte ratio' OR  'dnlr' OR 'lung immune prognostic index' OR 'lipi' OR 'peripheral blood biomarkers':ab,ti OR 'serum biomarkers':ab,ti OR 'predictive':ab,ti OR  'prognosis':ab,ti OR 'prognostic':ab,ti |
| #6 | #3 AND #4 AND #5 |
|  |  |
| **Cochrane Library** | 284 |
| #1 | "carcinoma, non small cell lung"[MeSH Terms] |
| #2 | (Carcinoma, Non-Small-Cell Lung):ti,ab,kw OR (Carcinoma, Non Small Cell Lung):ti,ab,kw OR (Carcinomas, Non-Small-Cell Lung):ti,ab,kw OR (Lung Carcinoma, Non-Small-Cell):ti,ab,kw OR (Lung Carcinomas, Non-Small-Cell):ti,ab,kw OR (Non-Small-Cell Lung Carcinomas):ti,ab,kw OR (Non-Small-Cell Lung Carcinoma):ti,ab,kw OR (Non Small Cell Lung Carcinoma):ti,ab,kw OR (Carcinoma, Non-Small Cell Lung):ti,ab,kw OR (Non-Small Cell Lung Carcinoma):ti,ab,kw OR (Non-Small Cell Lung Cancer):ti,ab,kw OR (Nonsmall Cell Lung Cancer):ti,ab,kw |
| #3 | #1 OR #2 |
| #4 | (immune checkpoint inhibitor):ti,ab,kw OR (ICI):ti,ab,kw OR (PD-1):ti,ab,kw OR (Programmed Cell Death 1 Protein):ti,ab,kw OR (PD-L1):ti,ab,kw OR (Programmed Cell Death 1 Receptor):ti,ab,kw OR (Immunotherapy):ti,ab,kw OR (pembrolizumab):ti,ab,kw OR (nivolumab):ti,ab,kw OR (atezolizumab):ti,ab,kw OR (durvalumab):ti,ab,kw OR (avelumab):ti,ab,kw OR (Cemiplimab):ti,ab,kw OR (Camrelizumab):ti,ab,kw OR (Sintilimab):ti,ab,kw OR (Tislelizumab):ti,ab,kw OR (Toripalimab):ti,ab,kw |
| #5 | (Lactate Dehydrogenase) OR (LDH) OR (derived neutrophil-to lymphocyte-ratio) OR (derived neutrophil-lymphocyte ratio) OR (dNLR) OR (Lung Immune Prognostic Index) OR (LIPI) OR (Peripheral Blood Biomarkers):ti,ab,kw OR (serum biomarkers):ti,ab,kw OR (Predictive):ti,ab,kw OR (Predictive):ti,ab,kw OR (prognosis):ti,ab,kw OR (prognostic):ti,ab,kw |
| #6 | #3 AND #4 AND #5 |


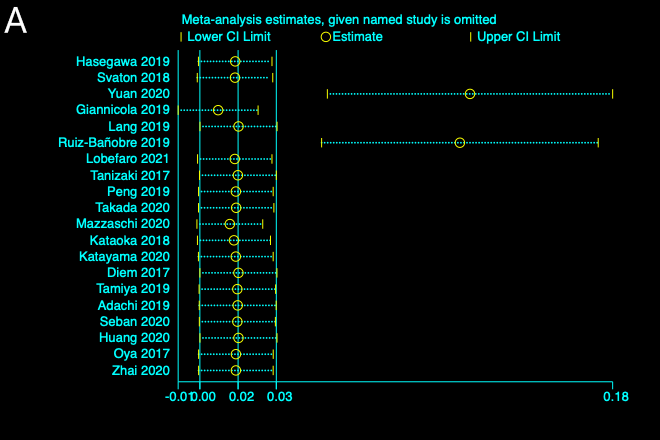


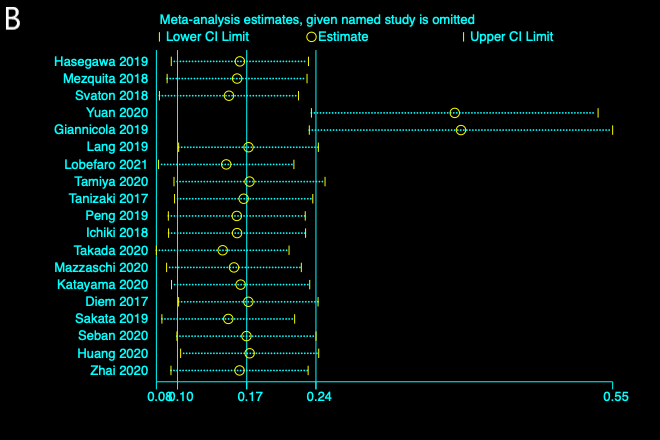


**Supplementary figure 1.** (A)Sensitivity analysis on the relationship between pretreatment LDH and PFS in advanced NSCLC; (B)Sensitivity analysis on the relationship between pretreatment LDH and OS in advanced NSCLC.


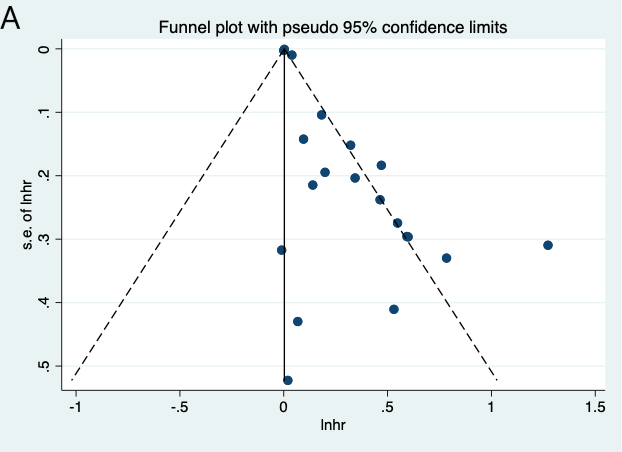

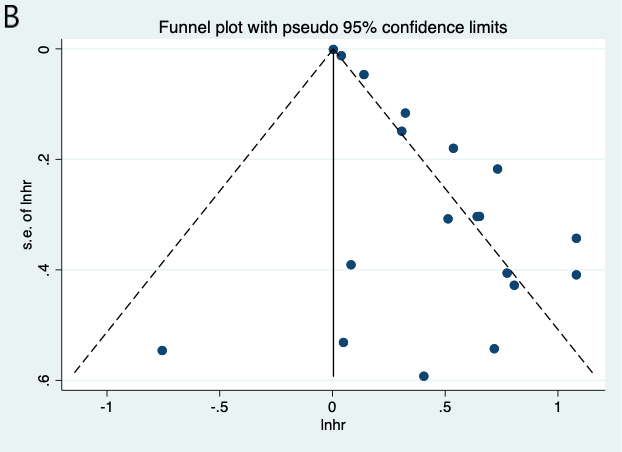


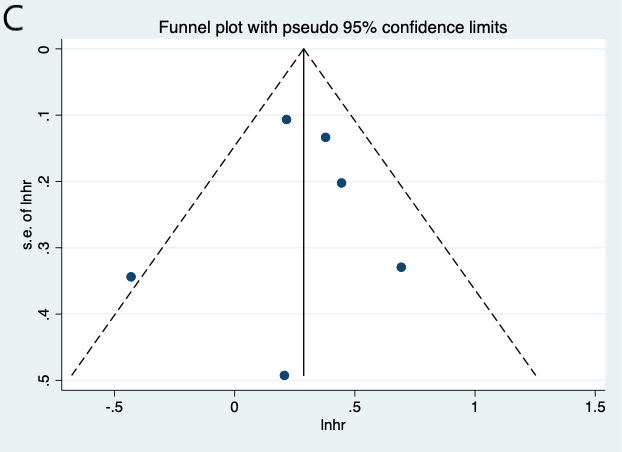

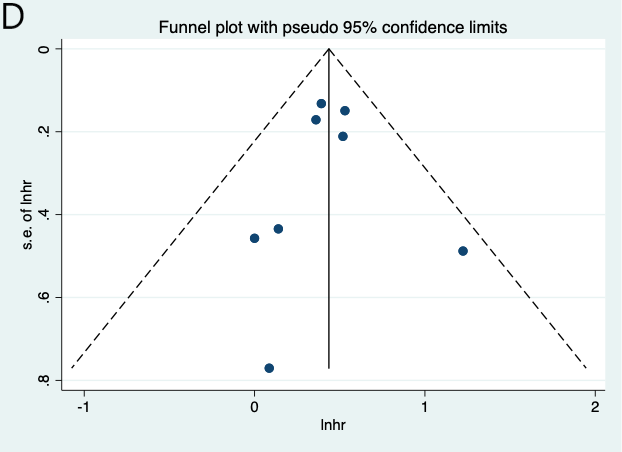


**Supplementary figure 2.** Funnel Plot of the relationship between(A) pretreatment LDH and PFS in advanced NSCLC,(B)pretreatment LDH and OS in advanced NSCLC,(C) pretreatment dNLR and PFS in advanced NSCLC,(D)pretreatment dNLR and OS in advanced NSCLC.


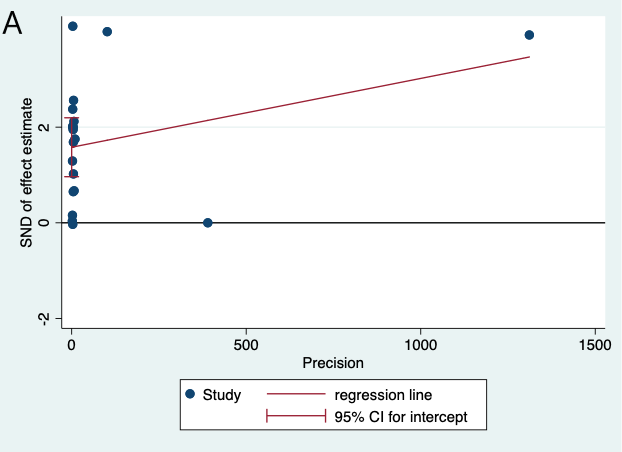

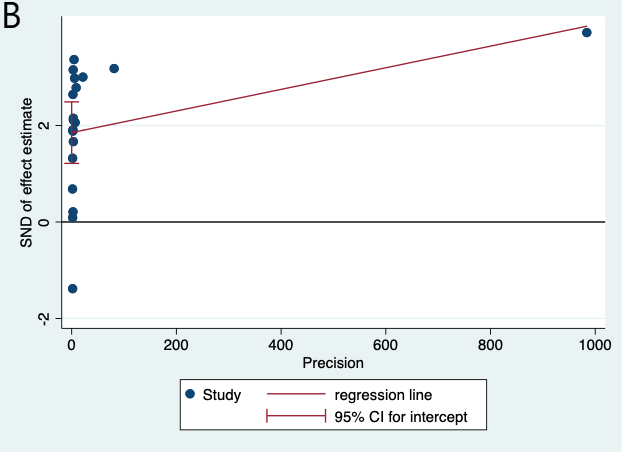


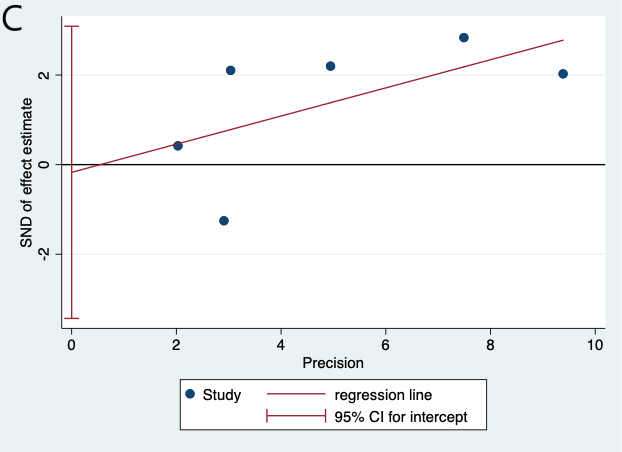

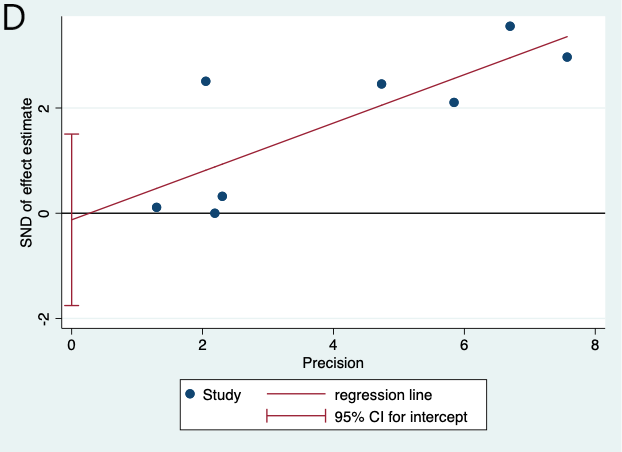


**Supplementary figure 3.** Egger’s test of the relationship between (A) pretreatment LDH and PFS in advanced NSCLC,(B)pretreatment LDH and OS in advanced NSCLC,(C) pretreatment dNLR and PFS in advanced NSCLC,(D)pretreatment dNLR and OS in advanced NSCLC.
